# Analysis of the ARTIC V4 and V4.1 SARS-CoV-2 primers and their impact on the detection of Omicron BA.1 and BA.2 lineage-defining mutations

**DOI:** 10.1099/mgen.0.000991

**Published:** 2023-04-21

**Authors:** Fatima R. Ulhuq, Madhuri Barge, Kerry Falconer, Jonathan Wild, Goncalo Fernandes, Abbie Gallagher, Suzie McGinley, Ahmad Sugadol, Muhammad Tariq, Daniel Maloney, Juliet Kenicer, Rebecca Dewar, Kate Templeton, Martin P. McHugh

**Affiliations:** ^1^​ Viral Genotyping Reference Laboratory, Royal Infirmary of Edinburgh, NHS Lothian, Edinburgh, EH16 4SA, UK; ^2^​ Institute of Evolutionary Biology, University of Edinburgh, Edinburgh, EH9 3JR, UK; ^3^​ School of Medicine, University of St Andrews, St Andrews, KY16 9TF, UK

**Keywords:** SARS-CoV-2, Nanopore, whole genome sequencing, Omicron, BA.1, BA.2

## Abstract

The ARTIC protocol uses a multiplexed PCR approach with two primer pools tiling the entire SARS-CoV-2 (severe acute respiratory syndrome coronavirus 2) genome. Primer pool updates are necessary for accurate amplicon sequencing of evolving SARS-CoV-2 variants with novel mutations. The suitability of the ARTIC V4 and updated V4.1 primer scheme was assessed using whole genome sequencing of Omicron from clinical samples using Oxford Nanopore Technology. Analysis of Omicron BA.1 genomes revealed that 93.22 % of clinical samples generated improved genome coverage at 50× read depth with V4.1 primers when compared to V4 primers. Additionally, the V4.1 primers improved coverage of BA.1 across amplicons 76 and 88, which resulted in the detection of the variant-defining mutations G22898A, A26530G and C26577G. The Omicron BA.2 sub-variant (VUI-22JAN-01) replaced BA.1 as the dominant variant by March 2022, and analysis of 168 clinical samples showed reduced coverage across amplicons 15 and 75. Upon further interrogation of primer binding sites, a mutation at C4321T [present in 163/168 (97 %) of samples] was identified as a possible cause of complete dropout of amplicon 15. Furthermore, two mutations were identified within the primer binding regions for amplicon 75: A22786C (present in 90 % of samples) and C22792T (present in 12.5 % of samples). Together, these mutations may result in reduced coverage of amplicon 75, and further primer updates would allow the identification of the two BA.2-defining mutations present in amplicon 75: A22688G and T22679C. This work highlights the need for ongoing surveillance of primer matches as circulating variants evolve and change.

## Introduction

On 11 March 2020, the World Health Organisation (WHO) declared a global pandemic following the emergence of the severe acute respiratory syndrome coronavirus 2 (SARS-CoV-2). To date, 5 September 2022, there have been over 600 million confirmed cases and over 6.5 million deaths worldwide [1].

Worldwide genomic surveillance of SARS-CoV-2 has been employed to better understand the viral mutation rate and transmission dynamics of emerging SARS-CoV-2 variants. Identifying the genetic diversity of circulating SARS-CoV-2 variants aids research into understanding the pathogenicity of variants, identifying sites on the genome that may reduce the effectiveness of vaccines and drug therapies, and ultimately inform the design of future therapeutics [2–8]. Additionally, real-time surveillance of SARS-CoV-2 outbreaks informs the implementation of control and prevention measures for SARS-CoV-2 transmission. As of 6 September 2022, 12.9 million SARS-CoV-2 genomes have been submitted to the global initiative on sharing all influenza data (GISAID) database (gisaid.org).

The most widely used protocol to sequence SARS-CoV-2 is the ARTIC amplicon-based targeted whole-genome sequencing approach. The ARTIC protocol is a freely available protocol allowing for rapid responses to changes in the SARS-CoV-2 viral genome [9–14]. This is a multiplex PCR approach, which consists of 98 pairs of primers that span the ~30 kb genome. However, one of the major limitations of the amplicon approach is the static nature of the primer scheme as the virus evolves and develops mutations and structural variants, which can lead to primer mismatches and an inability to construct near-complete viral genomes. Since January 2020 the primer scheme has been updated four times in response to changes in the prevailing virus lineages [9–11, 13, 14]. The inability of a given primer to bind to the complementary sequence results in amplicon dropouts, and mutations in the Beta, Delta and Gamma variants resulted in less efficient amplification of amplicons 72, 74 and 76 [14, 15].

Due to its high transmissibility rates, the Omicron variant BA.1 quickly became the dominant variant and was assigned as a variant of concern (VOC-21NOV-01). The large number of mutations present in the spike protein of BA.1 reduced the likelihood of yielding complete viral sequences. Analysis revealed that amplicons 76, 79 and 90 resulted in near-complete dropouts of the Omicron lineage BA.1 [13]. The original V4 primer scheme was updated with an additional 11 alternate primers (alts) to generate the V4.1 primers. Additional primers were added to amplify amplicons 10, 23, 27, 76, 79, 88, 89 and 90 in order to improve primer binding and eliminate amplicon dropouts for BA.1 [13].

In the presnt study, the suitability of the ARTIC V4.1 primer scheme by comparison with the V4 primer scheme is assessed and reported for the Omicron BA.1 lineage. To do this, Nanopore sequencing was used to sequence 70 SARS-CoV-2 samples, with both V4 and V4.1 primers, obtained from real-time reverse transcription (RT)-PCR confirmed COVID-19 patients. Subsequently, BA.2 was assigned as a variant under investigation (VUI-22JAN-01) and has increased in prevalence. The ability of the V4.1 primer scheme to generate near-complete genome coverage of BA.2 was also investigated in this work.

## Methods

### Patient samples and RNA extraction

In total, 344 SARS-CoV-2-positive clinical samples were collected between December 2021 and March 2022. There were 325 nose and throat swabs (94.48%), 18 throat swabs (5.23%) and one tracheal aspirate (0.29%). Samples were confirmed positive for at least two SARS-CoV-2 real-time RT-PCR targets using a range of diagnostic assays in NHS laboratories across the east of Scotland and referred to the Viral Sequencing Service, Royal Infirmary of Edinburgh, for sequencing. RNA extraction was performed on confirmed SARS-CoV-2 samples using two automated extraction platforms: the bioMérieux NucliSENS EMAG and Abbot *m2000sp*. First, SARS-CoV-2-positive samples were inactivated and lysed using NucliSENS easyMAG Lysis Buffer (bioMérieux, ref. 280134). When using the *m2000sp*, VTM and lysis buffer were mixed at a 1 : 1 ratio and equal volumes of diluted lysis buffer were added to each sample for lysis (500 µl lysis buffer +500 µl sample). For the bioMérieux NucliSens EMAG, neat lysis buffer was added to each sample (200 µl sample +2 ml lysis buffer). The procedure was followed as outlined in the manufacturer’s instructions and the RNA was eluted in 110 µl. VTM only was used as an internal control for each extraction run.

### ARTIC Lo-Cost Nanopore library preparation protocol

ONT libraries for SARS-CoV-2 sequencing were made according to the ARTIC Lo-Cost protocol [11, 12], with both V4 and V4.1 primers. Slight modifications to the published method were made during the DNA barcode ligation step. For a single reaction, the ligation mastermix was prepared by adding nuclease-free water (2.1 µl), Ultra II Ligation Mastermix (5.0 µl), Ligation Enhancer (0.2 µl) (NEBNext Ultra II Ligation Module, E7595S) and the unique barcode (1.2 µl) (ONT Native Barcoding Expansion 96, EXP-NBD196). The End Prep reaction mixture (1.5 µl), from the previous step, was added to the ligation mastermix (8.50 µl) containing the unique barcodes. The protocol was then followed as outlined in the ARTIC Lo-Cost protocol [11].

### Nanopore sequence analysis

High-accuracy basecalling was performed using Guppy (v4.4.0) (Oxford Nanopore Technologies) and demultiplexed using guppy_barcoder with the option ‘require_barcodes_both_ends’ and a minimum score of 60. Downstream analysis was performed using the ARTIC fieldbioinformatics pipeline (v1.2.1) (https://github.com/artic-network/fieldbioinformatics) and nanopolish variant calling (https://github.com/jts/nanopolish) to generate a consensus sequence for each sample in FASTA format. RAMPART (v1.0.6) (https://github.com/artic-network/rampart) was used to calculate genome coverage at 20×, 100× and 200× read depth. Nextclade (v1.10.3) (https://clades.nextstrain.org, dataset version 2022-03-14T12 : 00 : 00Z) was used to determine the number of SNPs between the reference genome (NC_045512.2) and sequenced clinical isolates. PANGOLIN (v3.1.20) (https://github.com/cov-lineages/pangolin) [16, 17] was used to assign a lineage to all SARS-CoV-2 samples, using the pangoLEARN (v1.2.124) algorithm and constellations (v0.1.3). Aln2type (v0.0.3) (https://github.com/connor-lab/aln2type) (analysis performed on 8 February 2022) was used to identify variant-defining mutations for VOC and VUI as curated by Public Health England (https://github.com/phe-genomics/variant_definitions).

The metrics and results of all experiments are available in the supplementary data (Datasets 1, 2 and 3). For direct V4 to V4.1 primer comparison, two separate 48-plex libraries were loaded and sequenced (Run1 and Run2, Dataset 1). For the analysis of BA.1 and BA.2 samples with V4.1 primers only, a total of seven 48-plex libraries were loaded and sequenced.

### Sequence alignment

Sequences were aligned to the reference SARS_CoV-2 isolate NC_045512.2 using MAFFT (v7.505) (https://anaconda.org/bioconda/mafft) [18] and visualized using Jalview2 (v2.11.2.2) [19]. Depth at each nucleotide position was extracted using SAMtools (v1.15) (https://github.com/samtools/samtools).

### Quality control

Samples were run as 48-plex libraries; each included a negative and positive control (SARS-CoV-2 B.1 lineage, collected June 2020) and two extraction negative controls (Datasets 1, 2 and 3). For all sequencing runs, the negative control and extraction negative controls generated fewer than five mapped reads and 0 % genome coverage at 20×. The viral lysate of the positive control generated 99.90 % genome coverage at 20× read depth. All seven expected mutations were detected for the positive control and assigned the PANGO lineage B.1. Clinical samples that generated <70 % whole genome coverage were not included in the comparison analysis detailed in this work.

### Data Summary

The data have been deposited with links to BioProject accession number PRJNA902683 in the NCBI BioProject database (http://www.ncbi.nlm.nih.gov/bioproject/902683).

#### Impact Statement

The most widely used protocol to sequence SARS-CoV-2 (severe acute respiratory syndrome coronavirus 2) is the ARTIC amplicon-based targeted whole-genome sequencing approach. The emergence of novel variants may result in poor amplicon sequencing and incomplete viral sequences. Therefore, the primer scheme needs to be continuously updated to respond to changes in the viral genome. The performance of the ARTIC V4 and V4.1 primer scheme was evaluated, and limitations with the current V4.1 primer scheme were identified. This work highlights the importance of ongoing monitoring of ARTIC primer schemes and the need for an updated primer scheme for accurate sequencing of circulating SARS-CoV-2 variants.

## Results

### Genome coverage of Omicron BA.1 with ARTIC V4 and V4.1 primers

In total, nine (13%) clinical samples were identified as Delta (VUI-21OCT-01 or VOC-21APR-02), 59 (84 %) clinical samples were identified as Omicron lineage BA.1 (VOC-21NOV-01) and two (3%) clinical samples were identified as Omicron BA.2 (VUI-22JAN-01) with ARTIC V4 and V4.1 primers (Dataset 1). For the Delta lineage samples, the median genome coverage at 20× read depth improved from 99.40 % with V4 primers to 99.90 % with V4.1 primers. All nine samples were confirmed as either VUI-21OCT-01 or VOC-21APR-02 with both V4 and V4.1 primers. The two Omicron BA.2 samples increased from 95.20– to 99.30% and from 97.20– to 99.90% genome coverage, with V4 and V4.1 primers, respectively. Both samples had probable aln2type VUI-22JAN-01 calls with V4 primers, but one sample improved from probable to confirmed with V4.1 primers.

Initial in-depth analysis focused on Omicron BA.1, as the increasingly dominant variant and the main component of the runs during sequencing. The genome coverage at >50× read depth was investigated to identify regions of the genome with reduced amplification. Nucleotide positions that generated <50× read depth in 15 % or more of the samples were considered as low-coverage regions. Coverage was compared between the V4 and V4.1 primer scheme. The V4.1 primer scheme included a new batch of primers added to the V4 primers to improve the coverage across amplicons 10, 23, 27, 76, 79, 88, 89 and 90 [13].

**Fig. 1.**
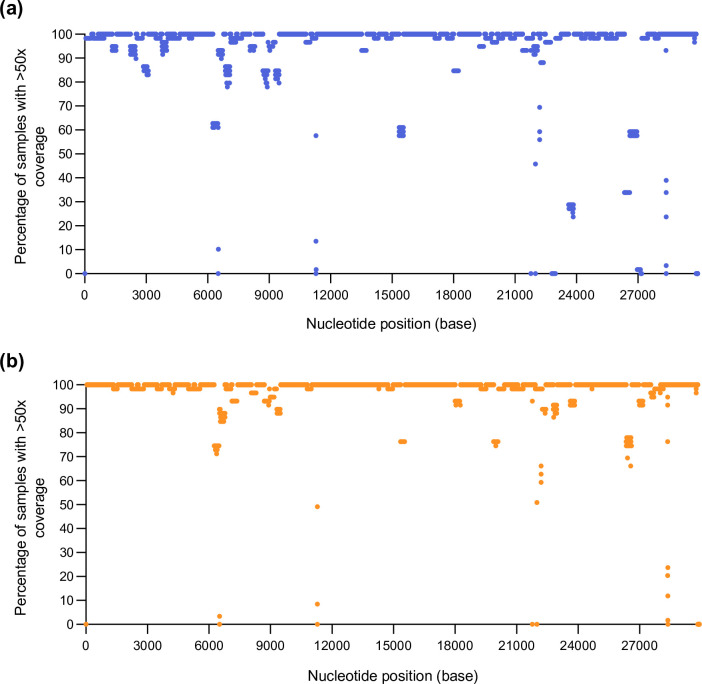
Coverage profile of SARS-CoV-2 BA.1 using V4 and V4.1 primers. Percentage of samples with >50× coverage at each nucleotide position for the assembly of 59 SARS-CoV-2 BA.1 samples with (**a)** V4 primers (in blue) or (**b)** V4.1 primers (in orange). The depth of each nucleotide position was generated using SAMtools on the primer-trimmed alignment files.

**Fig. 2.**
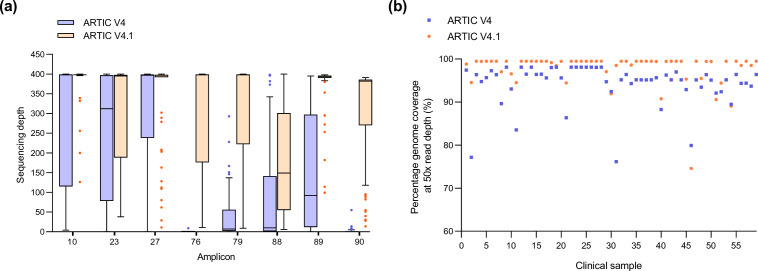
Increased sequencing depth across amplicons and increased genome coverage in SARS-CoV-2 BA.1 samples amplified with V4.1 primers. Analysis of 59 BA.1 samples with V4 and V4.1 primers.** (a)** Median coverage across amplicons 10, 23, 27, 76, 79, 88, 89 and 90 with V4 (blue) and V4.1 (orange). (**b)** Percentage genome coverage at >50× coverage of all 59 SARS-CoV-2 BA.1 samples. Assemblies with V4 primers are in blue, and V4.1 primers in orange.

Use of the V4 primers generated reduced amplification of nucleotides across 18 different amplicons, with less than 50× read depth in more than 15 % of sequenced samples (Fig. 1a, Table S1, available in the online version of this article). The use of the V4 primers resulted in dropout of amplicons 76 and 90 (Figs 1a and 2a, Table S1) and poor coverage across amplicons 10, 23, 79, 88 and 89 (Figs 1a and 2a, Table S1). Reduced coverage of these amplicons was due to primer mismatches between the V4 primer scheme and the SARS-CoV-2 BA.1 sequence. Reduced coverage of nucleotide positions across amplicons 22, 37, 72, 73, 74 and 95 was due to small deletions within the genome (3–9 bp) (Table S1). Reduced amplification of amplicons 21, 29, 31, 51 and 60 (Fig. 1a, Table S1) was detected with the V4 primers in samples that generated <60 000 mapped reads. Due to the low number of mapped reads for these samples, the reduced coverage within these amplicons may be due to the quality and quantity of extracted RNA from clinical samples, therefore, not likely to be due to primer mismatching and subsequent amplicon dropout.

Utilization of the V4.1 primer scheme [13] resulted in an overall improvement in genome coverage. The addition of the V4.1 alt primers increased the median sequencing depth across all eight amplicons with the modified primers in the updated V4.1 primer scheme (Fig. 2a). Strikingly, the additional primers rescued the dropout of amplicons 76 and 90 and increased sequencing coverage across amplicons 10, 23, 78, 88 and 89 (Fig. 2a). The number of amplicons with nucleotide positions that generated <50× coverage in 15 % or more samples decreased from 18 amplicons with V4 to 10 amplicons with V4.1 primers (Fig. 1b, Table S2). Despite the addition of modified primers to better amplify amplicon 88, the coverage defect was not completely restored. However, the percentage of sequenced samples with <50× coverage within this region decreased, from 40.68–66.1 % to 22.03–33.9 %, with V4 and V4.1 primers, respectively (Tables S1 and S2). Regions with lower coverage with the V4.1 primers were investigated to identify the root cause of lower read depth at certain nucleotide positions. The reduced coverage across amplicons 22, 37, 72, 73, 74 and 95 was due to small deletions within the genome (3–9 bp). Reduced amplification of amplicons 21, 51 and 66 was detected in samples with poor coverage across the entire genome due to sequences generating <60 000 mapped reads.

Analysis of all BA.1 sequences highlighted that, in total, 2668 nucleotides generated reduced coverage (<50× read depth in 15 % or more samples) with the V4 primers compared to 1085 nucleotides with the V4.1 primers (Tables S1 and S2). The median number of reads mapped per sample was 98 609 and 114 476 with V4 and V4.1 primers, respectively (Dataset 1). Additionally, the median number of masked bases (N’s) decreased from 1050 to 126 with the V4 and V4.1 primers, respectively (Dataset 1). In addition, the median number of SNPs increased from 52 to 58 with the V4 and V4.1 primers, respectively. This highlights the overall improvement detected with the V4.1 primers when compared to V4. The percentage of samples that generated improved genome coverage with the V4.1 primers when compared to V4 was 93.22 % (55/59) at 50× read depth (Fig. 2b). Additionally, 37 out of 59 (62.71 %) samples generated an increased number of mapped reads with the V4.1 primers compared to V4 (Dataset 1). The median genome coverage per sample for V4 primers at 200×, 100× and 20× was 95.59, 96.30 and 98.00 %, respectively (Dataset 1). The median coverage per genome with V4.1 primers at 200×, 100× and 20× was 98.80, 99.60 and 99.90 %, respectively.

### PANGO lineage and VOC/VUI calls of Omicron BA.1 with ARTIC V4 and V4.1 primers

PANGOLIN was used to assign a lineage to all SARS-CoV-2 consensus sequences, and 58/59 samples received identical PANGO lineages, either BA.1 (37/59) or BA.1.1 (21/59), with V4 and V4.1 primers (Dataset 1). The specificity of the lineage call for vssfru_032 altered from BA.1 to BA.1.1 with the V4.1 primers (vssfru_032b) when compared to V4 (vssfru_032b) (Dataset 1). Based on variant definitions by Public Health England, aln2type requires the detection of 17 variant-defining mutations to assign sequences as SARS-CoV-2 BA.1 (VOC-21NOV-01). All 17 variant-defining mutations must be detected for a ‘confirmed’ VOC/VUI status call. Interestingly, more accurate VOC-21NOV-01 status calls were obtained in 55/59 samples (93.22 %), improving from probable to confirmed VOC-21NOV-01 when using V4.1 compared to V4 primers (Dataset 1). The remaining 4/59 samples (6.78 %) had probable VOC-21NOC-01 calls with both V4 and V4.1 primers (Dataset 1).

**Fig. 3.**
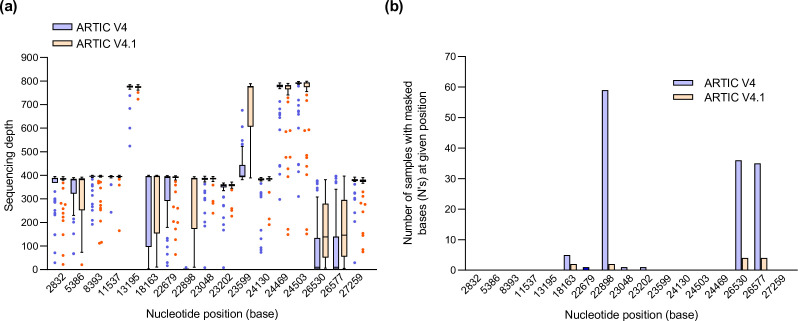
Increased sequencing depth at BA.1 variant-defining positions 22898, 26530 and 26577. Analysis of 59 BA.1 samples with V4 and V4.1 primers. (**a)** Sequencing depth of variant-defining mutations at all 17 nucleotide positions as defined by aln2type for BA.1 with V4 (blue) and V4.1 (orange) primers.** (b)** Number of samples with masked bases (N’s) at variant-defining nucleotide positions in BA.1 samples with V4 (blue) and V4.1 (orange) primers.

Further analysis highlighted that the median number of variant-defining mutations detected for VOC-21NOV-01 increased from 14 to 17 with the V4 and V4.1 primers, respectively. The increased coverage across amplicons 76 and 88 with the V4.1 primers (Fig. 2a) resulted in increased coverage at positions 22898, 26530 and 26577 (Fig. 3a) and the detection of the three additional variant-defining SNPs, G22898A, A26530G and C26577G, present in amplicons 76 and 88 (Fig. 3b). In addition, increased coverage of amplicon 60 allowed the identification of the variant-defining mutation A18163G (Fig. 3a, b). In total, 138 variant-defining bases were masked (N’s) with the V4 primers and this was reduced to 12 variant-defining bases with V4.1 primers (Fig. 3b). The 12 masked variant-defining bases at positions 18163 (*n*=2), 22898 (*n*=2), 26530 (*n*=4) and 26577 (*n*=4) (Fig. 3b) were confined to four BA.1 samples with poor genome quality and reduced coverage across the entire genome due to sequences generating <30 000 mapped reads.

A larger dataset of 106 positive SAR-CoV-2 BA.1 samples collected between February and March 2022 were investigated to compare the aln2type results obtained from the side-by-side V4 and V4.1 comparison above. Comparable results were obtained with reduced coverage at positions 18163, 22898, 26530 and 26577 (present in amplicons 60, 76 and 88) with the V4.1 primers (Fig. S1A, B). Reduced amplification of these regions contributed to only 16/106 (15.1 %) samples with probable VOC-21NOV-01 calls (Dataset 2) and a total of 48 masked variant-defining mutations (Fig. S1B, Dataset 2). Together, these results highlight the overall improvement in the amplification of Omicron BA.1 with the V4.1 primers when compared to V4.

### Genome coverage of Omicron BA.2 with ARTIC V4.1 primers

**Fig. 4.**
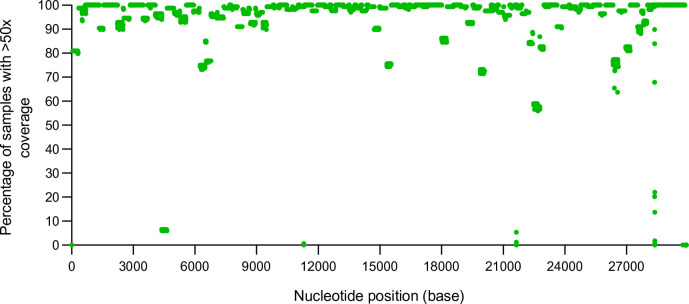
Coverage profile of SARS-CoV-2 BA.2 using V4.1 primers. Percentage of samples with >50× coverage at each nucleotide position for the assembly of 168 SARS-CoV-2 BA.2 samples with V4.1 primers (in green). The depth of each nucleotide position was generated using SAMtools on the primer-trimmed alignment files.

Genome coverage of Omicron BA.2 (VUI-22JAN-01) was further assessed with the ARTIC V4.1 primers by analysing 168 SARS-CoV-2-positive BA.2 samples collected between February and March 2022. Overall, the median genome coverage of 168 BA.2 samples with V4.1 primers at 200×, 100× and 20× was 96.15, 98.60 and 99.30 %, respectively (Dataset 3). The median number of mapped reads was 82 598 (Dataset 3). The median number of masked bases (N’s) was 387 with a median number of 68 SNPs across the BA.2 genomes. In total, 16 amplicons were identified with <50× read depth in more than 15 % of the samples sequenced (Fig. 4, Table S3). Reduced coverage of nucleotides across amplicons 37, 72, 95 and 99 was due to deletions within BA.2 (8–26 bp deletions). It was not possible to identify a cause for the reduced read depth of nucleotides present in amplicons 1, 21, 22, 51, 60, 66, 74, 76, 88 or 90, but BA.2 samples with reduced nucleotide coverage in these regions generated a total of <60 000 mapped reads per genome. This suggests that the low coverage may be due to pre-analytical issues with the amount and quality of RNA extracted from clinical samples. However, dropout of amplicon 15 and poor coverage across amplicon 75 was detected (Fig. 4, Table S3) in BA.2 samples with >60 000 mapped reads. These samples were further investigated to identify the root cause of reduced coverage.

**Fig. 5.**
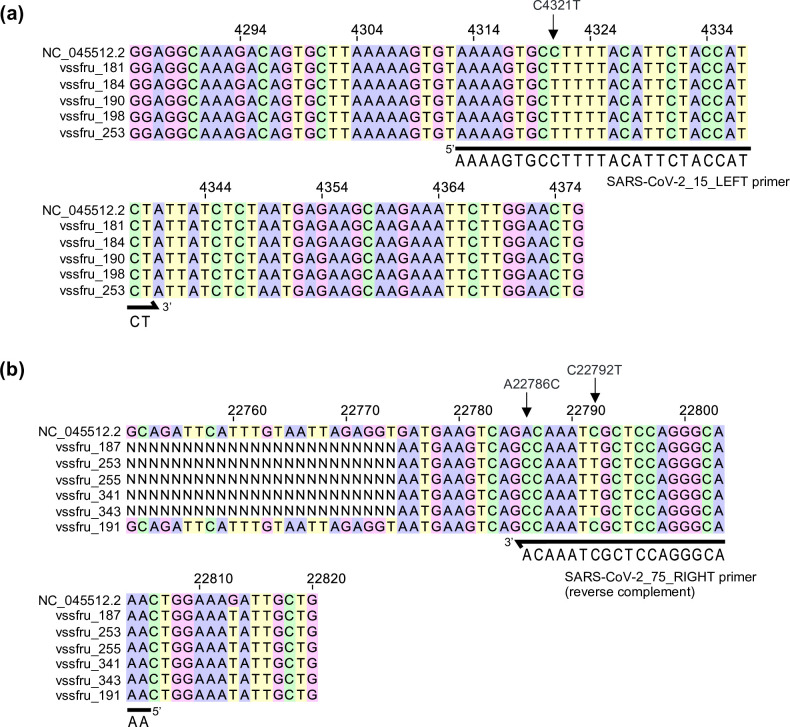
Identification of SNPs responsible for low coverage of amplicons 15 and 75 in BA.2 samples. Multiple sequence alignment using MAFFT (v7.505) of the NC_045512.2 reference with selected BA.2 samples with (**a)** reduced coverage of amplicon 15 or (**b)** reduced coverage of amplicon 75. Alignments were viewed using Jalview (v2.11.2.2).

With regard to amplicon 15, an SNP (C4321T) was identified in 163/168 samples (97 %) (Fig. 5a). The primer ‘SARS-CoV-2_15_LEFT’ binds within this region of the genome and the C4321T SNP sits 9 bp into the forward primer (Fig. 5a). In amplicon 75, two SNPs were identified within the primer binding region of ‘SARS-CoV-2_75_RIGHT’, A22786C and C22792T (Fig. 5b). The variant-defining mutation, A22786C, is present in 90 % of samples (151/168) while C22792T is present in 12.5 % of samples (21/168). The variant-defining mutation, A22786C, sits at the end of the ‘SARS-CoV-2_75_RIGHT’ primer (3′ region), and therefore does not have a significant impact on primer binding. However, samples with the C22792T SNP exhibit dropouts in amplicon 75, highlighting the importance of this site for primer binding (Fig. 5b). Updates to these primers may provide improved coverage of amplicons 15 and 75.

Reduced amplification of amplicons 75, 76 and 88 resulted in reduced sequencing coverage of four Omicron BA.2 (VUI-22JAN-01) variant-defining SNPs (T22679C, A22688G, A22786C and C26577G) (Fig. S2, Table S3). In total, 20 variant-defining mutations are required for a confirmed BA.2 (VUI-22JAN-01) status call, and 80 probable VUI-22JAN-01 calls were obtained with a total of 197 masked bases (N’s) at variant-defining positions across all BA.2 samples sequenced (Fig. S2B, Dataset 3). Of these, 71 BA.2 samples generated over 90 % genome coverage at 20× read depth and were then further investigated. The 71 probable VUI-22JAN-01 samples accumulated a total of 131 masked bases (N’s) at the following variant-defining positions: 2790 (*n*=3), 9424 (*n*=29), 18163 (*n*=7), 22679 (*n*=34), 22688 (*n*=34), 22775 (*n*=5), 22786 (*n*=9) and 26577 (*n*=10) (Dataset 3). As expected, masked bases (N’s) were detected at positions 22679, 22688, 22786 and 26577 due to lower coverage across amplicons 75, 76 and 88. However, the number of samples with masked bases at position 9424 (*n*=29) was unexpected, as 27 out of the 29 samples generated more than 20× coverage at position 9424 (Table S4). Further analysis revealed that the discrepancy in the detection of A9424G was due to the presence of mixed bases, with variant base calls often having low quality. This resulted in masking of the variant-defining SNP A9424G in our consensus calling pipeline due to low support for the variant call.

In addition, reference base calls at variant-defining sites, at either position 670 (6/168, 4 %) or 9866 (1/168, 0.6 %) (Table S5) contributed to probable VUI-22JAN-01 calls in seven BA.2 samples. This might be due to diversity within the BA.2 population, where reversion mutations may have occurred. Furthermore, a small cluster of 10 BA.2 samples were defined as having mixed calls for the variant-defining mutation at position 29510 (Table S5). An alignment comparing vssfru_253 (confirmed A29510C SNP), the SARS-CoV-2 reference genome (NC_045512.2) and the 10 BA.2 samples with aln2type mixed call status (Fig. S3) was generated (Fig. S3). The alignment highlights the presence of A29510C within all 10 BA.2 samples, but an additional SNP, G29511T, was present downstream (Fig. S3). The expected codon is CGT, but a subset of BA.2 samples have CTT within their consensus sequence, and this is then defined as a mixed call by aln2type. Strikingly, the presence of this unique mutation, not identified within any other sequences analysed within this work, may indicate transmission during a small outbreak.

## Conclusion

In summary, use of the ARTIC V4.1 primers, when compared to V4 primers, improved overall genome coverage of Omicron BA.1. Addition of the alt primers improved the sequencing depth across all eight amplicons, which resulted in more accurate PANGO lineage and VOC/VUI calls. Further analysis of the BA.2 sub-variant revealed complete drop-out of amplicon 15 and reduced coverage of amplicon 75. Additionally, lower read depth of amplicon 75 resulted in the masking of two variant-defining mutations. This work highlights the need for ongoing surveillance of primer matches as circulating variants evolve and change. The use of different primer schemes should be an important consideration when identifying and assigning VOC and the genome completeness of SARS-CoV-2 genomes should be routinely monitored with updates to primer schemes when required.

## Supplementary Data

Supplementary material 1

Supplementary material 2

Supplementary material 3

Supplementary material 4
