# Supplementary material for: Analysis of the ARTIC V4 and V4.1 SARS-CoV-2 primers and their impact on the detection of Omicron BA.1 and BA.2 lineage-defining mutations
Source: Microb Genom. 2023 Apr 21;9(4):mgen000991. doi: 10.1099/mgen.0.000991 (PMC10210939; doi:10.1099/mgen.0.000991)
Supplement: Supplementary material 1 [file mgen-9-991-s001.pdf]

Supplementary Information for

# Analysis of the ARTIC V4 and V4.1 SARS-CoV-2 primers and their impact on the detection of Omicron BA.1 and BA.2 lineage defining mutations

Fatima R. Ulhuq[1]*, Madhuri Barge[1], Kerry Falconer[1], Jonathan Wild[1], Goncalo Fernandes[1], Abbie Gallagher[1], Suzie McGinley[1], Ahmad Sugadol[1], Muhammad Tariq[1], Daniel Maloney[1,2], Juliet Kenicer[1], Rebecca Dewar[1], Kate Templeton[1], Martin McHugh[1,3]*

Corresponding authors:
Martin McHugh and Fatima Ulhuq

*To whom correspondence should be addressed.

e-mail: mpm20@st-andrews.ac.uk, fatima.ulhuq@nhslothian.scot.nhs.uk

**This PDF file includes:**

Tables S1 to S6
Figures S1 to S3

**Other supplementary materials for this manuscript include the following:**

Dataset 1
Dataset 2
Dataset 3

**Table S1.** Regions of the genome with <50x coverage in 15% or more BA.1 samples (n=59) sequenced using V4 primers .

| Amplicon | Nucleotides with <50x read depth (n) | Range of genomes with <50x read depth across nucleotide positions (%) | VOC-21NOV-01 defining mutations masked (N's) |
|---|---|---|---|
| 10 | 68 | 15.25 – 16.95% | NA |
| 21 | 260 | 37.29 – 38.98% | NA |
| 22 | 3 | 89.83 – 100% | NA |
| 23 | 77 | 15.25 – 22.03% | NA |
| 29 | 253 | 15.25 – 22.03% | NA |
| 31 | 191 | 15.25 – 20.34% | NA |
| 37 | 9 | 42.37 – 100% | NA |
| 51 | 221 | 38.98 – 42.37% | NA |
| 60 | 270 | 15.25% | A18163G |
| 72 | 5 | 100% | NA |
| 73 | 9 | 54.24 – 100% | NA |
| 74 | 3 | 30.51 – 44.07% | NA |
| 76 | 189 | 100% | G22898A |
| 79 | 265 | 71.19 – 76.27% | NA |
| 88 | 283 | 40.68 – 66.1% | A26530G<br>C26577G |
| 89 | 274 | 40.68 – 42.37% | NA |
| 90 | 282 | 40.68 – 100% | NA |
| 95 | 6 | 61.02 – 100% | NA |

**Table S2.** Regions of the genome with <50x coverage in 15% or more BA.1 samples (n=59) sequenced using V4.1 primers.

| Amplicon | Nucleotides with <50x read depth (n) | Range of genomes with <50x read depth across nucleotide positions (%) | VOC-21NOV-01 defining mutations masked (N's) |
|---|---|---|---|
| 21 | 260 | 25.42% – 28.8% | NA |
| 22 | 48 | 15.25 – 100 % | NA |
| 37 | 9 | 50.85 – 100% | NA |
| 51 | 221 | 23.73% | NA |
| 66 | 240 | 23.73 – 25.42% | NA |
| 72 | 5 | 100% | NA |
| 73 | 9 | 49.15 – 100% | NA |
| 74 | 3 | 33.9 – 40.68% | NA |
| 88 | 283 | 22.03 – 33.9% | A26530G<br>C26577G |
| 95 | 7 | 23.73 – 100% | NA |

**Table S3.** Regions of the genome with <50x coverage in 15% or more BA.2 samples (n=168) sequenced using V4.1 primers.

| Amplicon | Nucleotides with <50x read depth (n) | Range of genomes with <50x read depth across nucleotide positions (%) | VUI-22JAN-01 defining mutations masked (N's) |
|---|---|---|---|
| 1 | 294 | 19.05 – 20.24% | NA |
| 15 | 261 | 93.45 – 94.05% | NA |
| 21 | 260 | 25 – 27.79% | NA |
| 22 | 229 | 15.48 – 23.81% | NA |
| 37 | 9 | 99.40 – 100% | NA |
| 51 | 221 | 24.40 – 25.60% | NA |
| 60 | 6 | 15.48% | NA |
| 66 | 240 | 26.79 – 28.57% | NA |
| 72 | 9 | 94.64 – 100% | NA |
| 74 | 181 | 15.48 – 16.07% | NA |
| 75 | 300 | 41.07 – 44.05% | T22679C<br>A22688G |
| 76 | 189 | 17.26 – 18.45% | A22786C |
| 88 | 283 | 22.62 – 36.31% | C26577G |
| 90 | 221 | 17.26 – 19.05% | NA |
| 95 | 8 | 16.07 – 100% | NA |
| 99 | 26 | 100% | NA |

**Table S4.** Sequencing coverage of VUI-22JAN-01 samples with a masked base (no-call) at position A9424G (Dataset 3).

| Sample ID | Run no. | Barcode | Position | Reference base reads | Variant base reads | Status |
|---|---|---|---|---|---|---|
| vssfru_177 | Run3 | barcode01 | A9424G | 5 | 43 | no-call |
| vssfru_179 | Run3 | barcode14 | A9424G | 3 | 15 | no-call |
| vssfru_182 | Run3 | barcode20 | A9424G | 36 | 346 | no-call |
| vssfru_184 | Run3 | barcode30 | A9424G | 31 | 255 | no-call |
| vssfru_186 | Run3 | barcode33 | A9424G | 11 | 92 | no-call |
| vssfru_189 | Run3 | barcode38 | A9424G | 28 | 211 | no-call |
| vssfru_190 | Run3 | barcode39 | A9424G | 15 | 199 | no-call |
| vssfru_192 | Run3 | barcode45 | A9424G | 40 | 350 | no-call |
| vssfru_193 | Run4 | barcode49 | A9424G | 2 | 45 | no-call |
| vssfru_194 | Run4 | barcode51 | A9424G | 4 | 40 | no-call |
| vssfru_221 | Run5 | barcode23 | A9424G | 2 | 64 | no-call |
| vssfru_233 | Run6 | barcode53 | A9424G | 1 | 9 | no-call |
| vssfru_236 | Run6 | barcode59 | A9424G | 4 | 75 | no-call |
| vssfru_239 | Run6 | barcode75 | A9424G | 7 | 47 | no-call |
| vssfru_241 | Run6 | barcode79 | A9424G | 23 | 155 | no-call |
| vssfru_247 | Run6 | barcode92 | A9424G | 43 | 334 | no-call |
| vssfru_249 | Run7 | barcode01 | A9424G | 0 | 50 | no-call |
| vssfru_257 | Run7 | barcode11 | A9424G | 45 | 344 | no-call |
| vssfru_258 | Run7 | barcode12 | A9424G | 4 | 75 | no-call |
| vssfru_259 | Run7 | barcode13 | A9424G | 23 | 183 | no-call |
| vssfru_260 | Run7 | barcode14 | A9424G | 23 | 364 | no-call |
| vssfru_266 | Run7 | barcode25 | A9424G | 19 | 364 | no-call |
| vssfru_270 | Run7 | barcode31 | A9424G | 22 | 240 | no-call |
| vssfru_272 | Run7 | barcode35 | A9424G | 29 | 360 | no-call |
| vssfru_274 | Run7 | barcode37 | A9424G | 49 | 332 | no-call |
| vssfru_280 | Run7 | barcode48 | A9424G | 18 | 188 | no-call |
| vssfru_299 | Run8 | barcode72 | A9424G | 11 | 55 | no-call |
| vssfru_308 | Run8 | barcode84 | A9424G | 5 | 57 | no-call |
| vssfru_337 | Run9 | barcode37 | A9424G | 29 | 267 | no-call |

**Table S5.** Sequencing coverage of VUI-22JAN-01 samples with 1 reference call at a variant defining position as defined by aln2type (no-detect) (Dataset 3).

| Sample ID | Run no. | Barcode | Position | Reference base reads | Variant base reads | Status |
|---|---|---|---|---|---|---|
| vssfru_180 | Run3 | Barcode15 | T670G | 607 | 2 | no-detect |
| vssfru_233 | Run6 | Barcode53 | T670G | 580 | 9 | no-detect |
| vssfru_242 | Run6 | Barcode80 | T670G | 688 | 1 | no-detect |
| vssfru_245 | Run6 | Barcode89 | T670G | 769 | 6 | no-detect |
| vssfru_251 | Run7 | Barcode03 | T670G | 758 | 8 | no-detect |
| vssfru_261 | Run7 | Barcode15 | T670G | 764 | 6 | no-detect |
| vssfru_335 | Run9 | Barcode31 | C9866T | 385 | 2 | no-detect |

**Table S6.** Sequencing coverage of VUI-22JAN-01 samples with 1 mixed call (detect-mixed) at a variant defining position as defined by aln2type (Dataset 3).

| Sample ID | Run no. | Barcode | Position | Variant base | Sample-call | Status |
|---|---|---|---|---|---|---|
| vssfru_297 | Run8 | Barcode69 | A29510C | C | C,T | detect-mixed |
| vssfru_298 | Run8 | Barcode71 | A29510C | C | C,T | detect-mixed |
| vssfru_299 | Run8 | Barcode72 | A29510C | C | C,T | detect-mixed |
| vssfru_300 | Run8 | Barcode74 | A29510C | C | C,T | detect-mixed |
| vssfru_301 | Run8 | Barcode75 | A29510C | C | C,T | detect-mixed |
| vssfru_302 | Run8 | Barcode76 | A29510C | C | C,T | detect-mixed |
| vssfru_303 | Run8 | Barcode77 | A29510C | C | C,T | detect-mixed |
| vssfru_304 | Run8 | Barcode80 | A29510C | C | C,T | detect-mixed |
| vssfru_305 | Run8 | Barcode81 | A29510C | C | C,T | detect-mixed |
| vssfru_306 | Run8 | Barcode82 | A29510C | C | C,T | detect-mixed |

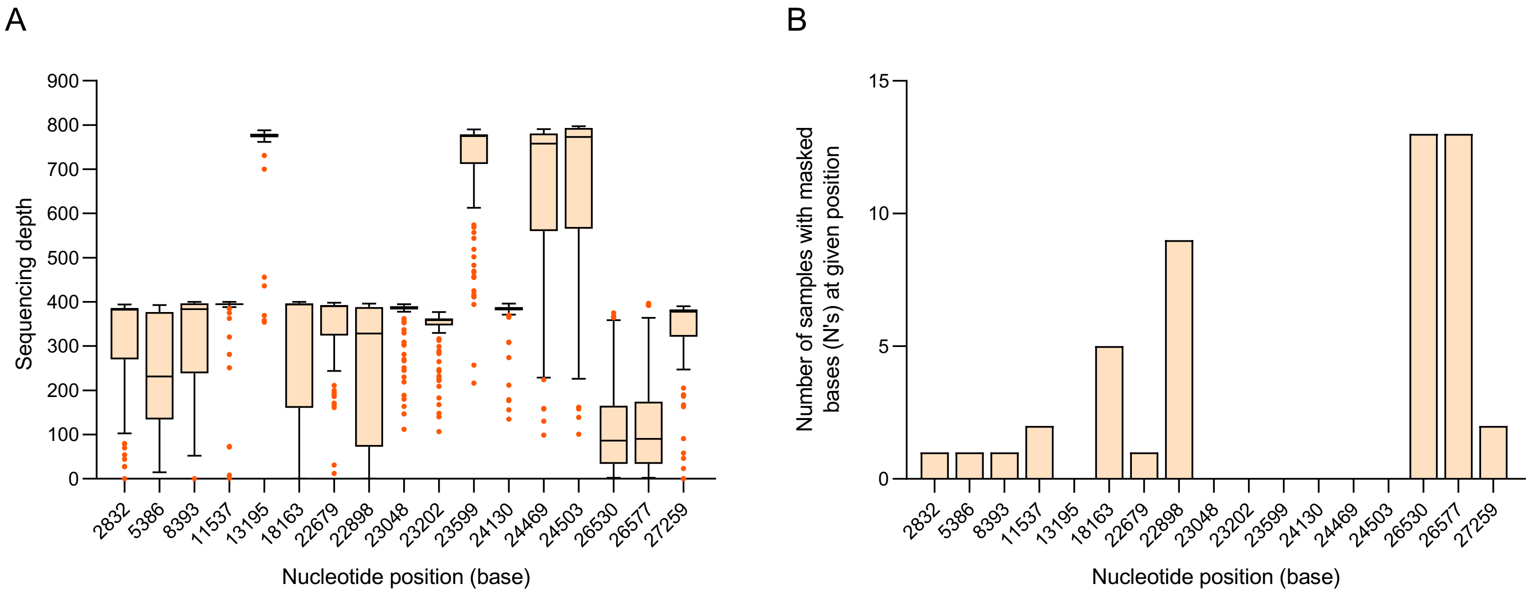


**Figure S1. Sequencing coverage and aln2type VOC-21NOV-01 defining mutations. Analysis of 106 BA.1 samples with V4.1 primers. A. Sequencing depth of variant defining mutations at all 17 nucleotide positions as defined by aln2type for BA.1. B. Number of samples with masked bases (N's) at variant defining nucleotide positions in BA.1 samples.**

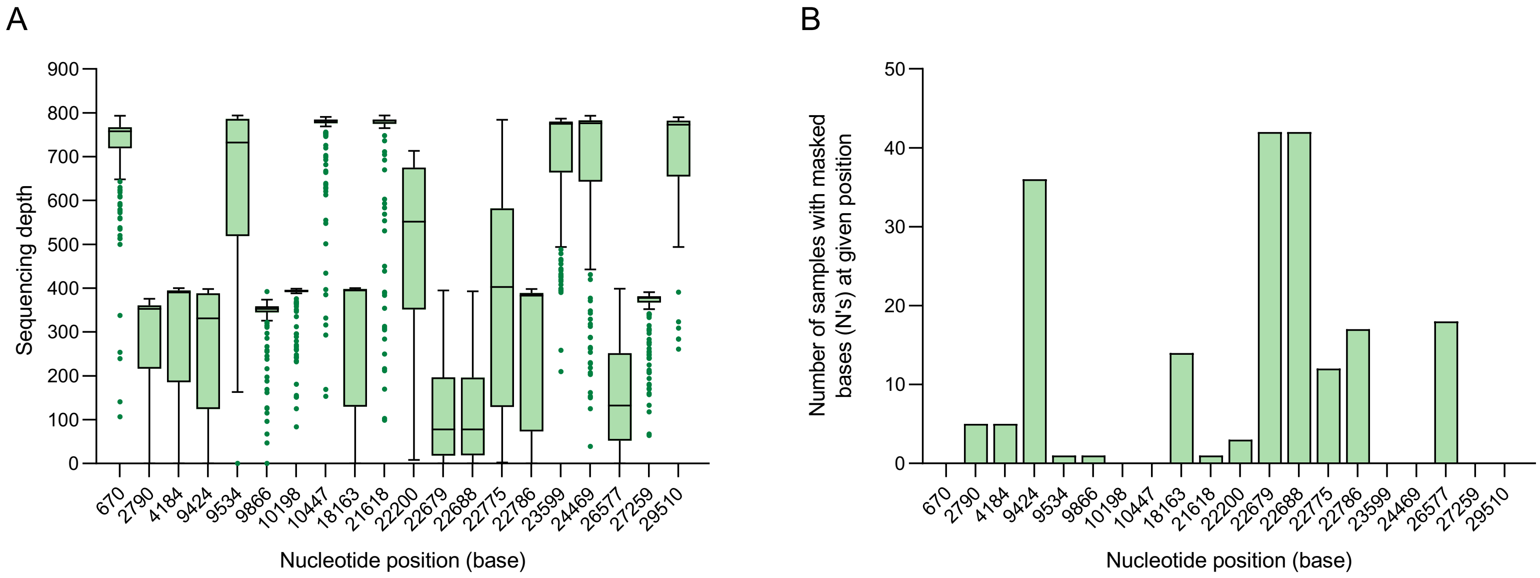


**Figure S2. Sequencing coverage and aln2type VUI-22JAN-01 defining mutations. Analysis of 168 BA.2 samples. A. Sequencing depth of variant defining mutations at all 20 nucleotide positions as defined by aln2type for BA.2. B. Number of samples with masked bases (N's) at variant defining nucleotide positions in BA.2 samples.**

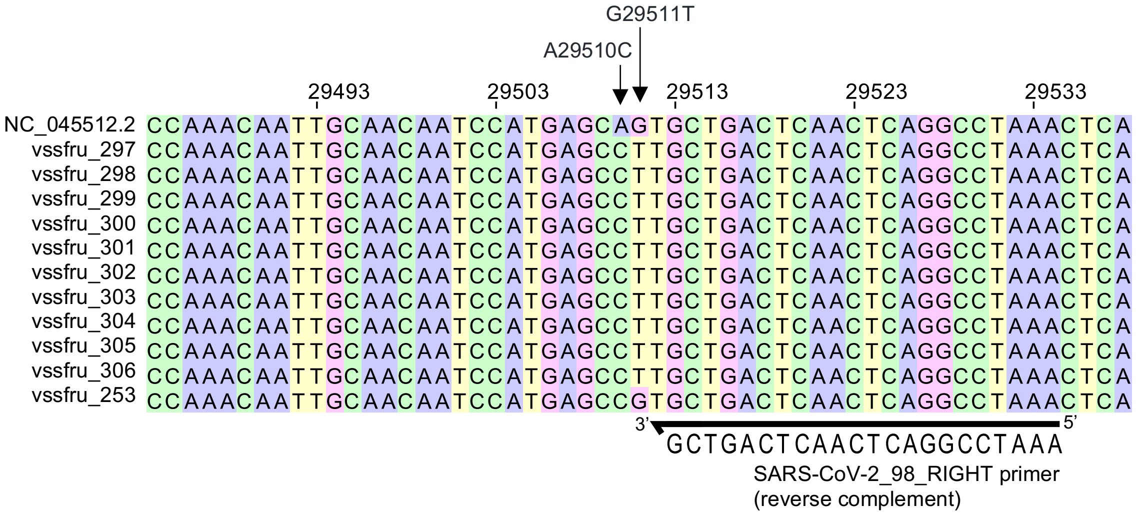


**Figure S3. A29510C aln2type mixed-call status due to downstream mutation present in subset of samples. Multiple sequence alignment using MAFFT (v7.505) of NC_045512.2 reference with 10 BA.2 samples with mixed-call status for A29510C variant defining mutation and vssfru_253 (A29510C detected). Alignment viewed using Jalview (v2.11.2.2).**
